# Correction: Lättman, K., et al. Perceived Accessibility, Satisfaction with Daily Travel, and Life Satisfaction among the Elderly. *Int. J. Environ. Res. Public Health* 2019, *16*, 4498

**DOI:** 10.3390/ijerph18084047

**Published:** 2021-04-12

**Authors:** Katrin Lättman, Lars E. Olsson, Margareta Friman, Satoshi Fujii

**Affiliations:** 1 CTF Service Research Center, Department of Social and Psychological Studies, Karlstad University, SE-65188 Karlstad, Sweden; katrin.lattman@kau.se (K.L.); lars.e.olsson@kau.se (L.E.O.); 2 Kyoto University, Katsura, Nishikyo-ku, Kyoto 615-8540, Japan; fujii@trans.kuciv.kyoto-u.ac.jp

## Text Correction

There were errors in the original article [1], and the authors would like to apologize for any inconvenience caused. The authors regret that due to a recently observed technical error in the mobile application used to collect survey data, biased data was observed for 528 out of 2950 respondents. New analyses with the smaller sample of 2422 respondents replicated the main findings, and most conclusions are still valid.

Some minor changes were observed. The explained variance (R2) changed from 0.05 to 0.17 for satisfaction with travel (STS) and from 0.18 to 0.14 for life satisfaction (LS). The reason for this is that technical errors were found in relation to these variables, which also resulted in increased mean scores of between 0.2 and 0.4 across age groups for both STS and LS in the new analyses. In the model, paths are now stronger from perceived accessibility (PAC) to both STS (from β = 0.22 to β = 0.39) and LS (from β = 0.15 to β = 0.31), and remain significant but less strong from STS to LS (from β = 0.36 to β = 0.13). Paths from car use to STS and from gender to PAC were slightly reduced. 

The previously observed moderation of age for the path between PAC and LS remains, although a slight change was observed in that the path was strongest for the old age groups, a finding explained by reduced travel and a shift in mode use when reaching the age of 75. For the paths between PAC and STS, and between STS and LS, moderation of age are no longer observed. Moderation for the path between PT use and PAC, and between being a women and PAC were replicated, while moderation of age related to car use and STS is no longer observed. Finally, comparisons of means scores for PAC, STS, and LS across the age brackets changed from being non-significant to significant. However, the effect sizes were negligible, ranging from 0.004 to 0.008.

In light of these new analyses, corrections have been made to several sections, such as the **Abstract, Method (2.1 Participants), Results (3.1 Travel mode Use; 3.2.1. Reliability and Validity of the Latent Constructs; 3.2.2. Testing the Structural Model; 3.2.3. Testing Moderation by Age Group; 3.2.4. Comparisons of Means across the Age Brackets for PAC, STS, and LS), 4. Discussion, and 5. Conclusions**.

The authors apologize for any inconvenience caused and state that the new analyses replicate the main findings, and that most of the scientific conclusions are still valid. 

The original article has been updated.

## Correction of Figure/Table Legend

As a consequence of the new analyses, the legend has been changed for **Figure 2**. by adding a note on marginally significant paths. The original article has been updated.

## Correction of Figure/Table

As a consequence of the new analyses, **Figure 2**., **Figure 3**., **Table 2**., **Table 5**., **Table 6**., and **Table 7**. have been changed. The original article has been updated.
